# Retracted: Intracoronary Adenosine versus Intravenous Adenosine during Primary PCI for ST-Elevation Myocardial Infarction: Which One Offers Better Outcomes in terms of Microvascular Obstruction?

**DOI:** 10.1155/2013/907848

**Published:** 2013-12-14

**Authors:** ISRN Cardiology

This article entitled “Intracoronary Adenosine versus Intravenous Adenosine during Primary PCI for ST-Elevation Myocardial Infarction: Which One Offers Better Outcomes in terms of Microvascular Obstruction?” [1], published in ISRN Cardiology, has been retracted upon the authors' request, as it was found to include erroneous data that their findings and conclusions cannot be relied upon. Additionally, the article was submitted for publication by the author Gemina Doolub without the knowledge and approval of the other author Erica Dall'Armellina.
